# Spatio-Temporal Field Estimation Using Kriged Kalman Filter (KKF) with Sparsity-Enforcing Sensor Placement

**DOI:** 10.3390/s18061778

**Published:** 2018-06-01

**Authors:** Venkat Roy, Andrea Simonetto, Geert Leus

**Affiliations:** 1 NXP Semiconductors, High Tech Campus 46, 5656 AE Eindhoven, The Netherlands; 2 Optimisation and Control group, IBM Research Ireland, Dublin 15, Ireland; andrea.simonetto@ibm.com; 3 Faculty of Electrical Engineering, Mathematics and Computer Science, Delft University of Technology, Mekelweg 4, 2628 CD Delft, The Netherlands; g.j.t.leus@tudelft.nl

**Keywords:** sparsity, kriging, Kalman filter, sensor placement, convex optimization

## Abstract

We propose a sensor placement method for spatio-temporal field estimation based on a kriged Kalman filter (KKF) using a network of static or mobile sensors. The developed framework dynamically designs the optimal constellation to place the sensors. We combine the estimation error (for the stationary as well as non-stationary component of the field) minimization problem with a sparsity-enforcing penalty to design the optimal sensor constellation in an economic manner. The developed sensor placement method can be directly used for a general class of covariance matrices (ill-conditioned or well-conditioned) modelling the spatial variability of the stationary component of the field, which acts as a correlated observation noise, while estimating the non-stationary component of the field. Finally, a KKF estimator is used to estimate the field using the measurements from the selected sensing locations. Numerical results are provided to exhibit the feasibility of the proposed dynamic sensor placement followed by the KKF estimation method.

## 1. Introduction

Tracking the spatio-temporal evolution of any field using a limited number of homogeneous/heterogeneous sensors with a desired accuracy is one of the most common applications of wireless sensor networks (WSNs) [1,2]. Different types of environmental, geophysical and biological processes exhibit complicated spatial as well as temporal variability. Spatial and temporal variability of a spatio-temporally stationary physical field can be modelled by its correlation over space and time [3]. If the field is non-stationary then a suitable dynamic model can be used to model the spatio-temporal evolution of the field [3]. If the field exhibits both a stationary and non-stationary behavior over space and time then the field can be dynamically monitored by the combination of kriging [3] and Kalman filtering, i.e., a kriged Kalman filter (KKF) [4] or space-time Kalman filter [5]. The key idea behind the KKF is the liaison of kriging [3] and Kalman filtering. The unknown physical field is modelled as a combination of a non-stationary (capturing the dynamics) and a stationary (capturing the low magnitude spatial effects) stochastic component. Assuming that the dynamics of the non-stationary component and the second-order statistics of the stationary component (e.g., covariance structure) are perfectly known, KKF jointly estimates both of these field components using the spatial observations at every time instant. The KKF paradigm has been used for different applications like wireless communications (e.g., spectrum sensing [6] and path delay estimation [7]) and field estimation [5]. From a practical perspective, KKF can also be used for air pollution level forecasting applications [8]. Also, a distributed implementation of KKF can be used for environment monitoring using a robotic sensor network [9].

One of the important overheads of dynamic field estimation using a WSN is the lack of sufficient measurements at every time instant. This is related to the shortage of sensor life time, availability of bandwidth, and other resource-related economical constraints. In such scenarios, we need to efficiently place/move the available sensors into the most informative locations over space and time. One notable approach for sensor placement in Gaussian processes is based on exploiting the submodularity of the mutual information between the sensing locations [10]. Submodularity of the frame potential and related greedy algorithms for sensor placement are proposed in [11]. Dynamic sensor scheduling is a well-cultivated topic in the fields of signal processing as well as control theory  [12,13]. Some recent notable contributions are [14,15,16]. Prior knowledge regarding the correlation of the field over space and time can be exploited in a multi-layer design of sensor networks [17]. Selecting the most informative sensing locations can be treated as a sensor selection problem, which can be formulated as a convex optimization problem [18]. This can be solved for linear [19] as well as non-linear measurement models [20]. Sparsity-promoting approaches for sensor placement are also exhibited in [15,21], where the placement algorithm is formulated using the alternating direction method of multipliers (ADMM). In [22], a generalized sparsity-enforcing and performance-constrained sensor placement method is proposed, where the field can be either stationary or non-stationary. The aforementioned method can be implemented for a single snapshot or multiple snapshot ahead sensor placement and for a general class of spatio-temporal covariance matrices, which can either be ill-conditioned or well-conditioned. Seminal contributions on the convex formalism of sensor selection (like [18]) assume that the measurement noise components are spatio-temporally uncorrelated. However, this can be an unrealistic assumption in some practical scenarios [23]. However, even in those scenarios, it has been shown that the sensor selection problem can be formulated as a convex optimization problem [16,24].

In this work, we develop a unified framework of sensor placement followed by a KKF estimator to dynamically monitor a physical field that exhibits both stationarity and non-stationarity over space and time. In the first step, we select the most informative locations to deploy/move the sensors and in the second step we estimate the field by using the measurements from those selected locations. The key contributions can be summarized as follows,

- The performance metrics to estimate the stationary as well as the non-stationary components of the field are represented in closed form as an explicit function of the sensor location selection vector.
- The aforementioned analytical formalism tackles two important issues in the sensor placement step. First, the developed method takes care of the fact that the estimation of the non-stationary component of the field involves the stationary component of the field as a spatially correlated observation noise. Second, the proposed method is applicable for a general class of spatial covariance matrices of the stationary component of the field, even when they are ill-conditioned or close to singular [25].
- The proposed sensor placement problem is formulated in a way that minimizes a cost function that involves the sum of the mean square error (MSE) of the stationary and the non-stationary component of the field as well as a spatial sparsity enforcing penalty. The overall optimization problem also satisfies a flexible resource constraint at every time instant.

One of the aspects that distinguishes the proposed sensor placement method from the prior works in sensor placement for environmental field estimation is the specific statistical nature of the unknown physical field, which yields an additive coupling of stationary and the non-stationary components. Secondly, we develop a unified framework for the efficient utilization of the spatio-temporal variability of the field in order to design an opportunistic sensor placement method using a convex approach. We develop a parsimonious sensor placement algorithm followed by a KKF estimator, which can be used to dynamically monitor a general class of environmental fields (based on the assumed process model and spatial statistics of the field components). However, the developed approach is similar to [22] in terms of the primary measurement model, which is considered to be linear and underdetermined. We emphasize that the proposed technique is a model-based centralized sensor placement method, where we resort to the Bayesian estimation philosophy. We assume that the available prior statistical knowledge regarding the unknown physical field like spatial correlation information and dynamics are perfectly known a priori. It is also assumed that for the current centralized setup the communication range of the sensors are sufficient to communicate with the fusion center, which can be located inside/outside the given service area.

*Notations*: Matrices are in upper case bold while column vectors are in lower case bold. The notation ${\left[X\right]}_{ij}$ is the (i,j)-th entry of the matrix X, ${\left[x\right]}_{i}$ is the *i*-th entry of the vector x, and tr[X] denotes the trace of X, i.e., the sum of the diagonal elements of X. The notation supp(x) is defined as the set of indices of the non-zero entries of x, while diag(x) and diag(X) are the diagonal matrix with diagonal x and the main diagonal of the matrix X, respectively. An identity matrix of size N×N is denoted by $I_{N}$. The notation ${\left(\cdot \right)}^{T}$ is the transpose operator, $\hat{x}$ is the estimate of x, and ${∥x∥}_{p}={\left(\sum_{i=0}^{N-1}{\left|{\left[x\right]}_{i}\right|}^{p}\right)}^{1/p}$ is the $\ell_{p}$ norm of x. Vectors of all zeros and ones of length *N* are denoted by $0_{N}$ and $1_{N}$, respectively. An all zero matrix of size N×N is given by $0_{N\times N}$. The set of symmetric matrices of size N×N and the set of symmetric positive-definite matrices of size N×N are denoted by $S^{N}$ and $S_{++}^{N}$, respectively.

## 2. Signal Modelling and Problem Formulation

### 2.1. Measurement Model

Let us denote the spatially continuous field by $u_{t}\left(x\right)$, at any discrete time index *t* and location $x\in R^{2}$. We assume that the entire service area of interest is *uniformly discretized* over *N* pixels, where we would like to estimate the field intensities. The field intensities of the *N* pixels at time *t* are represented by $u_{t}\in R^{N}$. It is assumed that the field intensity is the same everywhere within a pixel, and it can be represented by ${\left[u_{t}\right]}_{j}=u_{t}\left(x_{j}\right)$, where $x_{j}\in R^{2}$ is the centroid of the *j*-th pixel, with j=1,…,N. We consider a linear underdetermined measurement model

(1) $$\begin{array}{cc} y_{t} & =C_{t}u_{t}+e_{t} \end{array}$$

(2) $$\begin{array}{c} =C_{t}\left(v_{t}+s_{t}\right)+e_{t}, \end{array}$$

where $v_{t}\in R^{N}$ is the non-stationary component of the field and $s_{t}\in R^{N}$ is a stationary component of the field capturing the non-dynamic spatial effects. It is assumed that $v_{t}$ and $s_{t}$ are mutually uncorrelated.

At any time *t*, the measurements are given by $y_{t}\in R^{M_{t}}$ collected from $M_{t}$ ($M_{t}<N$) sensing locations (pixels) of the entire service area. The time-varying sensing or measurement matrix $C_{t}\in {\left\{0,1\right\}}^{M_{t}\times N}$ selects $M_{t}$ measurements from *N* field locations. The measurement matrix $C_{t}$ is constructed by removing the zero rows of $\mathrm{diag}(w_{t})$, where $w_{t}\in {\left\{0,1\right\}}^{N}$ is a *sensor location selection* vector. If ${\left[w_{t}\right]}_{j}=1\left(0\right)$, then the *j*-th pixel is selected (not selected) for sensor placement. Based on this, at any time *t*, the number and the constellation of the selected sensing locations are given by $1^{T}w_{t}=M_{t}$ and $\mathrm{supp}(w_{t})$, respectively. Using the considered construction of $C_{t}$, we have the relations

(3) $$C_{t}C_{t}^{T}=I_{M_{t}};\;\;C_{t}^{T}C_{t}=\mathrm{diag}\left(w_{t}\right).$$

Note that the type of measurement matrix used in this work is similar to an incidence matrix, which can be viewed as a flexible data collection method using heterogeneous sensing equipments. In practice, when different types of sensing modalities are used, we may not know the process by which any of the sensors gathers its measurement but only its recorded value is important. Also, we rigorously exploit the property of the structure of $C_{t}$ mentioned in (3), later in this paper.

The error incurred by the measurement process is modelled through $e_{t}$, which is uncorrelated with both $v_{t}$ and $s_{t}$, respectively. The spatio-temporally white measurement noise $e_{t}$ is characterized by $e_{t}∼N\left(0_{M_{t}},\sigma_{e}^{2}I_{M_{t}}\right)$.

### 2.2. Modelling of the Spatial Variability

**Figure 1:**
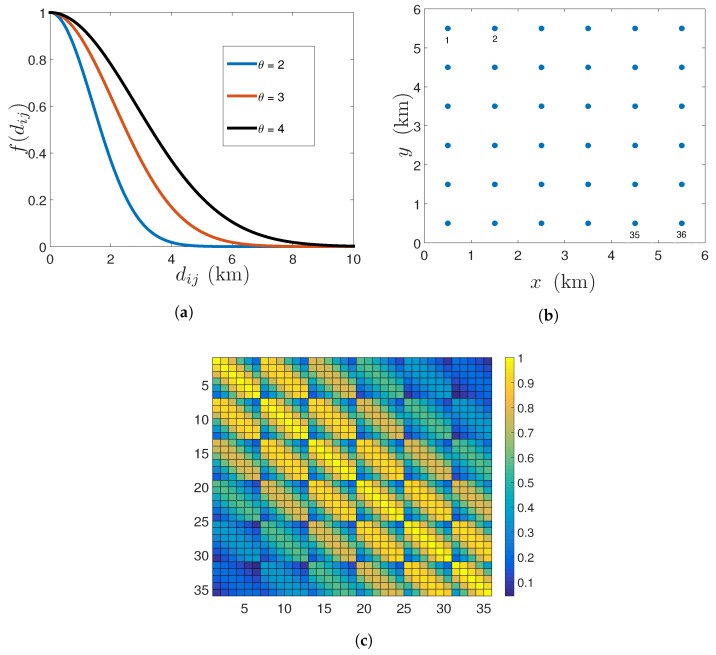
(**a**) Squared exponential covariance function for different values of θ (variance $\sigma_{s}^{2}=1$); (**b**) Service area with N=36 candidate sensing locations; (**c**) Spatial covariance matrix.

The spatial effects of the field are modelled through a spatially colored yet temporally white discrete random process $s_{t}∼N\left(\mu_{s},\Sigma_{s}\right)$, where $\mu_{s}\in R^{N}$ is the mean and $\Sigma_{s}\in S_{++}^{N}$ is the spatial covariance matrix of $s_{t}$. We assume that the process $s_{t}$ is spatially second-order stationary as well as isotropic, which means that

(4) $$\begin{array}{c} \;\mu_{s}=E\left[s_{t}\right]=\mu_{s}1_{N}, \end{array}$$

(5) $$\begin{array}{cc} {}[\Sigma_{s}{]}_{ij} & =E[\left(s_{t}\left(x_{i}\right)-\mu_{s}\right)\left(s_{t}\left(x_{j}\right)-\mu_{s}\right)]=f(∥x_{i}-x_{j}{∥}_{2}) \end{array}$$

where i,j=1,…,N [3]. Note that here we follow the same spatial discretization as mentioned in Section 2.1. There are several empirical as well as parametric model-based approaches to model the spatial covariance. In this work, we assume that the spatial covariance function is given by a simple squared exponential function:

(6) $$f(∥x_{i}-x_{j}{∥}_{2})=\sigma_{s}^{2}\exp\left(-\frac{∥x_{i}-x_{j}{∥}_{2}^{2}}{\theta^{2}}\right),$$

where θ>0 is the parameter controlling the strength of the spatial correlation. The covariance function mentioned in (6) is plotted in Figure 1a for increasing values of the pairwise distance between the centroids of the pixels, i.e., $d_{ij}={∥x_{i}-x_{j}∥}_{2}$ and the parameter θ. Note that the aforementioned covariance function belongs to the family of Matérn covariance functions [3], which are widely used to model the spatial variability of a field in geostatistics and environmental sciences.

Using the squared exponential covariance function, the elements of the N×N spatial covariance matrix ($\Sigma_{s}$) can be constructed by the Relation (5). Let us consider a service area with N=36 pixels. The centroids of these 36 pixels, which are also the candidate locations for sensor deployment are shown in Figure 1b. These centroids are indexed from the top left to the bottom right. The elements of $\Sigma_{s}$ are shown in Figure 1c. Note that based on the nature of the covariance Function (6), the spatial covariance matrix $\Sigma_{s}$ is symmetric and based on the constellation of the candidate sensing locations (Figure 1b), $\Sigma_{s}$ is also a block Toeplitz matrix. We assume that $\mu_{s}$ and $\Sigma_{s}$ are perfectly known a priori.

### 2.3. State Model

The spatio-temporal evolution of the non-stationary component of the field, i.e., $v_{t}$, can be modelled by the following state model

(7) $$v_{t}=H_{t}v_{t-1}+q_{t}.$$

Here, the time-varying state transition/propagator matrix is given by $H_{t}\in R^{N\times N}$. The process noise vector $q_{t}$ is assumed to be characterized by $q_{t}∼N\left(0,Q_{t}\right)$. The elements of the state transition matrix $H_{t}$ act as spatial redistribution weights for $v_{t-1}$ for the temporal transition from t−1 to *t* [3]. Note that this spatial redistribution can be dependent on the temporal sampling interval. We model the elements of $H_{t}$ by using a parameterized Gaussian kernel function

(8) $${\left[H_{t}\right]}_{ij}=\nu \;\exp\left[-{\left(x_{i}-x_{j}-a_{t}^{ij}\right)}^{T}{\left[D_{t}^{ij}\right]}^{-1}\left(x_{i}-x_{j}-a_{t}^{ij}\right)\right],$$

where i,j=1,…,N and the spatio-temporally varying translation and dilation parameters are represented by $a_{t}^{ij}\in R^{2}$, and $D_{t}^{ij}\in S_{++}^{2}$, respectively. The scalar ν∈(0,1) is a scaling parameter to avoid an explosive growth of $v_{t}$, i.e., to keep the maximum eigenvalue of $H_{t}$ below 1. The simplest form of the state model (7) is defined by $H_{t}\approx \nu I_{N}$, which is similar to a Gaussian random walk model. This corresponds to the Gaussian kernel Function (8), with $D_{t}=\zeta I$ and ζ≪1, and with $a_{t}=0$. In this work, we assume that the state transition matrix $H_{t}$, whose elements are parameterized by $\left\{a_{t}^{ij}\right\}$ and $\left\{D_{t}^{ij}\right\}$ through the Function (8) is perfectly known a priori.

From a practical point of view, the aforementioned Gaussian kernel-based spatio-temporal evolution can be used for rainfall prediction [26]. This modelling approach incorporates some physical properties of environmental fields, such as diffusion, advection etc. [26], so it can also be used for modelling the propagation of a general class of environmental fields (e.g., pollutants, aerosol movements).

### 2.4. Main Problem Statement

The main problem is to design an optimal sensor placement pattern, i.e., to design $w_{t}$, whose support gives the optimal locations to deploy the sensors. At any *t*, the design goal is to minimize the estimation error for both the stationary and the non-stationary components of the field as well as to enforce sparsity in $w_{t}$, i.e., to reduce the number of required sensing locations. If the estimation error of the stationary and non-stationary components of the field can be represented by a single performance metric $g(w_{t})$, the sensor placement problem can be represented by

(9a) $$\begin{array}{c} \underset{w_{t}\in {\left\{0,1\right\}}^{N}}{arg min}\;g\left(w_{t}\right)+\lambda_{t}{∥w_{t}∥}_{1} \end{array}$$

(9b) $$\begin{array}{c} \;s.t.\;K_{t}^{\min}\leq 1^{T}w_{t}\leq K_{t}^{\max}. \end{array}$$

At any *t*, $K_{t}^{\min}$ and $K_{t}^{\max}$ denote the lower bound on the number of available sensors, and a given budget on the maximum number of available sensors, respectively. Sparsity is enforced through a sparsity-promoting penalty, i.e., an $\ell_{1}$ norm of $w_{t}$ in the second summand of the cost Function (9a) with a time-varying regularization parameter $\lambda_{t}>0$ controlling the sparsity of the elements of $w_{t}$. A detailed description regarding the structure of the objective function and the importance of the constraints in the optimization problem (9) are discussed in Section 3.1.

### 2.5. Simple KKF Estimator and Estimation Error Covariance

Using the measurement and state models of (1) and (7), respectively, the state estimate ${\hat{u}}_{t}$, for t=1,2,…, can be computed following the lines of a standard KKF [5,6]. First, a simple Kalman filter is used to track the dynamic component $v_{t}$, where the stationary component $s_{t}$ is interpreted as a noise term. In this case, the measurement model is given by

(10) $$\begin{array}{c} {\overset{˘}{y}}_{t}=C_{t}v_{t}+{\overset{˘}{e}}_{t}, \end{array}$$

where ${\overset{˘}{y}}_{t}=y_{t}-C_{t}\mu_{s}$, ${\overset{˘}{e}}_{t}=C_{t}{\overset{˘}{s}}_{t}+e_{t}$, and ${\overset{˘}{s}}_{t}=s_{t}-\mu_{s}$. Furthermore, ${\overset{˘}{s}}_{t}∼N\left(0_{N},\Sigma_{s}\right)$, and ${\overset{˘}{e}}_{t}∼N\left(0_{M_{t}},{\overset{˘}{R}}_{t}\right)$, with ${\overset{˘}{R}}_{t}=C_{t}\Sigma_{s}C_{t}^{T}+\sigma_{e}^{2}I_{M_{t}}$. It can be easily shown that $v_{t}$ and ${\overset{˘}{e}}_{t}$ are mutually uncorrelated as it is already assumed in Section 2.1 that $v_{t}$ is mutually uncorrelated with $s_{t}$ and $e_{t}$, respectively. Now, using the state model of (7) and the measurement model of (10), the non-stationary component $v_{t}$ can be estimated following the lines of a simple Kalman filter [27]. In this case, the recursive state estimate at time *t* is given by

(11) $${\hat{v}}_{t}=H_{t}{\hat{v}}_{t-1}+G_{t}\left({\overset{˘}{y}}_{t}-C_{t}H_{t}{\hat{v}}_{t-1}\right),$$

where the Kalman gain $G_{t}$ can be expressed as

(12) $$\begin{array}{cc} G_{t}=[H_{t}M_{v_{t-1}}H_{t}^{T} & +Q_{t}]C_{t}^{T}\times \\ & {\left[{\overset{˘}{R}}_{t}+C_{t}\left(H_{t}M_{v_{t-1}}H_{t}^{T}+Q_{t}\right)C_{t}^{T}\right]}^{-1}. \end{array}$$

The MSE matrix of the estimate ${\hat{v}}_{t}$ at time *t* is given by $E\left[\left(v_{t}-{\hat{v}}_{t}\right){\left(v_{t}-{\hat{v}}_{t}\right)}^{T}\right]=M_{v_{t}}$, which is related to the MSE matrix of the estimate at time t−1, i.e., $M_{v_{t-1}}$, by the recursive relation [27]

(13) $$M_{v_{t}}={\left[{\left(H_{t}M_{v_{t-1}}H_{t}^{T}+Q_{t}\right)}^{-1}+C_{t}^{T}{\overset{˘}{R}}_{t}^{-1}C_{t}\right]}^{-1}.$$

In the next stage, the estimate of ${\hat{v}}_{t}$ in (11) is used to compute the stationary component $s_{t}$ using kriging. In spatial statistics, the intensity of an environmental field in an unknown location can be interpolated using a variogram model [3,4]. For a spatially stationary field, this variogram can be expressed as a covariance [3]. In this way kriging can be viewed as a simple linear minimum mean square error (LMMSE) interpolator [27]. The linear model is given by $y_{t}-C_{t}{\hat{v}}_{t}=C_{t}s_{t}+e_{t}$ and the related estimator has the form

(14) $${\hat{s}}_{t}=\mu_{s}+\Sigma_{s}C_{t}^{T}{\left(C_{t}\Sigma_{s}C_{t}^{T}+\sigma_{e}^{2}I_{M}\right)}^{-1}\left(y_{t}-C_{t}{\hat{v}}_{t}-C_{t}\mu_{s}\right),$$

where we use the prior information $s_{t}∼N\left(\mu_{s},\Sigma_{s}\right)$. The MSE matrix [27] of the estimate ${\hat{s}}_{t}$, i.e., $M_{s_{t}}$ is given by

(15) $$M_{s_{t}}={\left[\Sigma_{s}^{-1}+\sigma_{e}^{-2}C_{t}^{T}C_{t}\right]}^{-1}.$$

Finally, the overall field estimate at time *t* is given by ${\hat{u}}_{t}={\hat{v}}_{t}+{\hat{s}}_{t}$.

### 2.6. Performance Metrics as a Function of $w_{t}$

In this section, we express the MSE matrices, i.e., $M_{v_{t}}$ and $M_{s_{t}}$ as functions of $w_{t}$. First of all, we mention some facts regarding the structure of the error covariance matrices presented in the Expressions (13) and (15).

It should be noted that the measurement noise in (10) is correlated over space through the off-diagonal elements of ${\overset{˘}{R}}_{t}$. Due to this fact, sensor selection approaches using the standard convex framework like [18,20,22], i.e., designing a $w_{t}$ by directly optimizing the Expression (13) is difficult due to the presence of the off-diagonal elements of ${\overset{˘}{R}}_{t}$. It should also be noted that the expression of ${\overset{˘}{R}}_{t}$ is a function of the measurement matrix $C_{t}$ and thus the selection vector $w_{t}$. However, we do not encounter this issue in the performance metric to estimate the stationary component $s_{t}$, i.e., (15), as the measurement noise $e_{t}$ is assumed to be spatially uncorrelated in this case.

**Figure 2:**
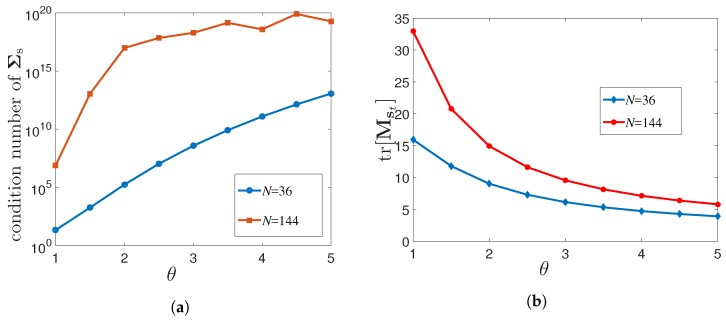
(**a**) Plot of the condition number of $\Sigma_{s}$ vs. θ with different number of candidate sensing locations (*N*); (**b**) MSE of the estimate of $s_{t}$ vs. θ for different numbers of candidate sensing locations (*N*); $M_{t}=N$; $\sigma_{e}^{2}=1$; The spatial resolution is increased by representing one pixel of Figure 1a by 4 pixels.

In the expression of $M_{s_{t}}$, i.e., (15), we assume that $\Sigma_{s}$ is well-conditioned, i.e., accurately invertible. However, this may not be the case in some scenarios. The condition number of $\Sigma_{s}$ strongly depends on the correlation of the field, spatial sampling distance, grid size etc. [25]. The variation of the condition number of $\Sigma_{s}$ with different values of θ for both N=36 and N=144 is plotted in Figure 2a. It is seen that for a higher resolution or a strong spatial correlation, the spatial covariance matrix becomes increasingly ill-conditioned and thus close to singular. In such circumstances, we cannot compute the estimation error covariance matrix $M_{s_{t}}$ using the Expression (15). In that case, $M_{s_{t}}$ can be computed using the alternate expression of (15) given by

(16) $$\begin{array}{cc} M_{s_{t}} & ={\left[\Sigma_{s}^{-1}+\sigma_{e}^{-2}C_{t}^{T}C_{t}\right]}^{-1}\\ & =\Sigma_{s}-\Sigma_{s}C_{t}^{T}{\left(C_{t}\Sigma_{s}C_{t}^{T}+\sigma_{e}^{2}I_{M_{t}}\right)}^{-1}C_{t}\Sigma_{s}, \end{array}$$

which is obtained using the matrix inversion lemma (MIL). It should be noted that the alternative expression of the MSE can be used to compute the MSE (without inverting $\Sigma_{s}$), but it is difficult to express it as an explicit function of $w_{t}$.

In Figure 2b, we plot $\mathrm{tr}[M_{s_{t}}]$ for the best case, i.e., the MSE with all the pixel centroids equipped with sensors ($w_{t}=1_{N}$ or $C_{t}=I_{N}$) for different values of θ, and for two different spatial resolutions (N=36 and N=144) of the same 6×6 square km service area. It is seen that $\mathrm{tr}[M_{s_{t}}]$ decreases as the strength of the correlation is increased by increasing θ.

To circumvent the effect of ill-conditioning as well as to handle the correlated measurement noise in the expression of $M_{v_{t}}$, we propose the following approach. We start by introducing the substitution

(17) $$\Sigma_{\mathrm{sr}}=\Sigma_{s}+\alpha I,$$

where $\Sigma_{\mathrm{sr}}$ is a well-conditioned matrix and α∈R. More specifically, the range of α is considered to be $0<\alpha <\sigma_{e}^{2}$. Substituting $\Sigma_{s}=\Sigma_{\mathrm{sr}}-\alpha I$, we can represent the measurement error covariance matrix of (10) as, ${\overset{˘}{R}}_{t}=C_{t}\Sigma_{\mathrm{sr}}C_{t}^{T}+\zeta I_{M_{t}}$, where $\zeta =\sigma_{e}^{2}-\alpha$ and where we used $C_{t}C_{t}^{T}=I_{M_{t}}$. Substituting ${\overset{˘}{R}}_{t}=C_{t}\Sigma_{\mathrm{sr}}C_{t}^{T}+\zeta I_{M_{t}}$ in (13), the MSE matrix for the estimate of the non-stationary component is given by

(18) $$\begin{array}{cc} M_{v_{t}}=[(H_{t}M_{v_{t-1}}H_{t}^{T} & +Q_{t}{)}^{-1}+\\ & C_{t}^{T}{\left(C_{t}\Sigma_{\mathrm{sr}}C_{t}^{T}+\zeta I_{M_{t}}\right)}^{-1}C_{t}{]}^{-1}. \end{array}$$

Using the MIL, we have the following matrix identity

(19) $$\begin{array}{cc} (\Sigma_{\mathrm{sr}}^{-1}+C_{t}^{T}(\zeta I_{M_{t}} & {)}^{-1}C_{t}{)}^{-1}=\Sigma_{\mathrm{sr}}\\ & -\Sigma_{\mathrm{sr}}C_{t}^{T}{\left(C_{t}\Sigma_{\mathrm{sr}}C_{t}^{T}+\zeta I_{M_{t}}\right)}^{-1}C_{t}\Sigma_{\mathrm{sr}}, \end{array}$$

from which we can derive

(20) $$\begin{array}{cc} C_{t}^{T}(C_{t}\Sigma_{\mathrm{sr}}C_{t}^{T}+\zeta I_{M_{t}} & {)}^{-1}C_{t}=\Sigma_{\mathrm{sr}}^{-1}\\ & -\Sigma_{\mathrm{sr}}^{-1}{\left(\Sigma_{\mathrm{sr}}^{-1}+\zeta^{-1}\mathrm{diag}\left(w_{t}\right)\right)}^{-1}\Sigma_{\mathrm{sr}}^{-1}, \end{array}$$

where we used $C_{t}^{T}C_{t}=\mathrm{diag}\left(w_{t}\right)$. Substituting (20) in (18) we obtain the following expression for $M_{v_{t}}$.

(21) $$\begin{array}{cc} M_{v_{t}}=[(H_{t}M_{v_{t-1}} & H_{t}^{T}+Q_{t}{)}^{-1}+\Sigma_{\mathrm{sr}}^{-1}\\ & -\Sigma_{\mathrm{sr}}^{-1}{\left(\Sigma_{\mathrm{sr}}^{-1}+\zeta^{-1}\mathrm{diag}\left(w_{t}\right)\right)}^{-1}\Sigma_{\mathrm{sr}}^{-1}{]}^{-1}. \end{array}$$

Next, substituting $\Sigma_{s}=\Sigma_{\mathrm{sr}}-\alpha I$ in the inverse of the right most term of (16) and using $C_{t}C_{t}^{T}=I_{M_{t}}$, we obtain

(22) $$M_{s_{t}}=\Sigma_{s}-\Sigma_{s}C_{t}^{T}{\left(C_{t}\Sigma_{\mathrm{sr}}C_{t}^{T}+\zeta I_{M_{t}}\right)}^{-1}C_{t}\Sigma_{s}.$$

Substituting the identity (20) into (22), we obtain the following expression of $M_{s_{t}}$.

(23) $$\begin{array}{cc} M_{s_{t}}=\Sigma_{s} & -\Sigma_{s}\Sigma_{\mathrm{sr}}^{-1}\Sigma_{s}\\ & +\Sigma_{s}\Sigma_{\mathrm{sr}}^{-1}{\left(\Sigma_{\mathrm{sr}}^{-1}+\zeta^{-1}\mathrm{diag}\left(w_{t}\right)\right)}^{-1}\Sigma_{\mathrm{sr}}^{-1}\Sigma_{s}. \end{array}$$

Note that, the expression of (23) avoids the inversion of an ill-conditioned $\Sigma_{s}$. Here, we only need to invert the well-conditioned $\Sigma_{\mathrm{sr}}$.

In this work, we consider the overall MSE as a performance metric for sensor placement, i.e., $g(w_{t})$ as mentioned in (9a). This is given by

(24) $$\begin{array}{cc} g(w_{t}) & =\mathrm{tr}\left(M_{v_{t}}\right)+\mathrm{tr}\left(M_{s_{t}}\right)\\ & =\mathrm{tr}{\left[X-F{\left[F+\zeta^{-1}\mathrm{diag}\left(w_{t}\right)\right]}^{-1}F\right]}^{-1}+\mathrm{tr}\left[Y\right]\\ & +\mathrm{tr}[Z^{T}{\left[F+\zeta^{-1}\mathrm{diag}\left(w_{t}\right)\right]}^{-1}Z], \end{array}$$

where $X={\left(H_{t}M_{v_{t-1}}H_{t}^{T}+Q_{t}\right)}^{-1}+\Sigma_{\mathrm{sr}}^{-1}$, $F=\Sigma_{\mathrm{sr}}^{-1}$, $Y=\Sigma_{s}-\Sigma_{s}\Sigma_{\mathrm{sr}}^{-1}\Sigma_{s}$, and $Z=\Sigma_{\mathrm{sr}}^{-1}\Sigma_{s}$. Note that the matrices X, F, Y, and Z are all independent of $w_{t}$. To model $\Sigma_{\mathrm{sr}}$ and $F+\zeta^{-1}\mathrm{diag}\left(w_{t}\right)$ as positive definite matrices we need $0<\alpha <\sigma_{e}^{2}$.

The performance metric derived in (24) incorporates the MSE matrices of the estimates of the non-stationary ($v_{t}$) as well as the stationary ($s_{t}$) component of the field, as explicit functions of the sensor location selection vector $w_{t}$. Note that a formulation similar to (23), for the computation of the MSE matrix as a function of $w_{t}$ is proposed in [22], where the field is considered to be either purely stationary or non-stationary.

## 3. KKF with Sensor Placement

In this section, we relax and reformulate the proposed sensor placement problem (9) as a semidefinite programming (SDP) problem. Then we present the overall KKF estimator followed by the sensor placement to dynamically monitor the field using only the measurements from the selected sensing locations.

### 3.1. Sensor Placement Problem as an SDP

Solving for the best subset of sensing locations is a combinatorially complex problem. However, it can be relaxed to a convex problem [18,19,20]. As discussed in Section 2.1, the sensor location selection vector $w_{t}\in {\left\{0,1\right\}}^{N}$ acts as a weighting vector for all the *N* candidate pixels. Following the main optimization problem, i.e., (9), a sparsity-enforcing, low estimation error, and resource-constrained design of $w_{t}$ can be obtained by solving

(25a) $$\begin{array}{c} \underset{w_{t}\in {\left[0,1\right]}^{N}}{arg min}\;g\left(w_{t}\right)+\lambda_{t}{∥w_{t}∥}_{1} \end{array}$$

(25b) $$\begin{array}{c} \;s.t.\;K_{t}^{\min}\leq 1^{T}w_{t}\leq K_{t}^{\max}, \end{array}$$

where the expression of $g(w_{t})$ is given by (24). Here, we have relaxed the non-convex Boolean constraint $w_{t}\in {\left\{0,1\right\}}^{N}$ of (9) to a convex box constraint $w_{t}\in {\left[0,1\right]}^{N}$. The resource constraint of (25b) is affine and thus convex. Some comments regarding the formulation of the proposed sensor placement problem of (25) are presented next.

- First of all, let us consider the non-convex version of the optimization problem of (25) with $\lambda_{t}=0$. This is given as
  (26a) $$\begin{array}{c} \underset{w_{t}\in {\left\{0,1\right\}}^{N}}{arg min}\;g\left(w_{t}\right) \end{array}$$
  (26b) $$\begin{array}{c} \;s.t.\;K_{t}^{\min}\leq 1^{T}w_{t}\leq K_{t}^{\max}. \end{array}$$
  In this case, the MSE cost will be minimum, i.e., the best estimation performance is achieved, when we select the maximum number of available candidate locations or in other words, when $1^{T}w_{t}=K_{t}^{\max}$. Then, there is no way to reduce the number of selected locations below $K_{t}^{\max}$ and the constraint $1^{T}w_{t}\geq K_{t}^{\min}$ becomes redundant. In the aforementioned case, it is difficult to reduce the number of selected sensing locations below $K_{t}^{\max}$.
- Notice that, dropping the resource constraint (25b) and increasing $\lambda_{t}$ will reduce the number of selected sensing locations. However, there is no explicit relation between $\lambda_{t}$ and $1^{T}w_{t}$, i.e., it is difficult to directly control the resource allocation (i.e., $K_{t}^{\max}$) through $\lambda_{t}$.
- We mention that the proposed formulation of (25) is not a direct MSE minimization problem but it attains a specific MSE along with enforcing sparsity in spatial sensor location selection through the second summand of (25a). The sparsity enforcement is lower bounded by the minimum number of sensing locations to be selected at any *t*, i.e., $K_{t}^{\min}$. It should be noted that for an arbitrary selection of $\lambda_{t}$, the minimum number of selected sensing locations will always be $K_{t}^{\min}$.
- Lastly, it should be noted that a sparsity-enforcing design of $w_{t}$ can be achieved by retaining only the second summand of the objective function of (25a) and using a separate performance constraint given as $g\left(w_{t}\right)\leq \gamma_{t,\mathrm{MSE}}$ [20,22]. The desired performance threshold $\gamma_{t,\mathrm{MSE}}$ can be time-varying or independent of *t* based on the application. However, in many practical scenarios, it could be difficult to set the performance threshold $\gamma_{t,\mathrm{MSE}}$ a priori for every *t*.

Based on the aforementioned arguments, we advocate the proposed design approach (25) that lowers the MSE along with enforcing sparsity in sensor placement satisfying a flexible resource allocation constraint.

After solving (25), we obtain ${\hat{w}}_{t}\in {\left[0,1\right]}^{N}$ which can be converted to a Boolean selection vector $w_{t}\in {\left\{0,1\right\}}^{N}$. This can be performed by either deterministic or stochastic rounding procedures as discussed below.

- The simplest approach could be to set the non-zero entries of ${\hat{w}}_{t}$ to 1. However, there can be a huge difference between the magnitudes of any two non-zero elements in ${\hat{w}}_{t}$. Considering the fact that the indices of the high magnitude (close to 1) elements of ${\hat{w}}_{t}$ signify a more informative sensing location, ${\hat{w}}_{t}$ can be sorted in ascending order of magnitude [18] and a selection threshold (γ) can be selected based on the magnitudes of the elements of the sorted ${\hat{w}}_{t}$. The entries of the Boolean selection vector can be computed as ${\left[w_{t}\right]}_{j}=1$ if ${\left[{\hat{w}}_{t}\right]}_{j}\geq \gamma$ else ${\left[w_{t}\right]}_{j}=0$, for j=1,…,N.
- Another approach could be a stochastic approach, where every entry of ${\hat{w}}_{t}$ is assumed to be the probability that this sensing location is selected at time *t*. Based on this, multiple random realizations of $w_{t}\in {\left\{0,1\right\}}^{N}$ are generated, where the probability that ${\left[w_{t}\right]}_{j}=1$ is given by ${\left[{\hat{w}}_{t}\right]}_{j}$, for j=1,…,N. Then the realization that satisfies the constraints and minimizes the estimation error, i.e., $g(w_{t})$ is selected [20].

Let us now transform the optimization problem of (25) into an SDP. From the expression of (24), it is clear that minimizing $g(w_{t})$ w.r.t. $w_{t}$ is equivalent to minimizing the expression $\mathrm{tr}{\left[X-F{\left[F+\zeta^{-1}\mathrm{diag}\left(w_{t}\right)\right]}^{-1}F\right]}^{-1}+\mathrm{tr}\left[Z^{T}{\left[F+\zeta^{-1}\mathrm{diag}\left(w_{t}\right)\right]}^{-1}Z\right]$ as the matrix tr[Y] is independent of $w_{t}$. In the first step, we represent the optimization problem of (25) in an epigraph form ([28], p. 134), ([18], Equations (25) and (26)) which is given by

(27a) $$\begin{array}{c} \underset{w_{t}\in {\left[0,1\right]}^{N},V\in S^{N},B\in S^{N}}{arg min}\;\;\mathrm{tr}\left[V\right]+\mathrm{tr}\left[B\right]+\lambda_{t}{∥w_{t}∥}_{1} \end{array}$$

(27b) $$\begin{array}{c} \;s.t.\;V⪰{\left[X-F{\left[F+\zeta^{-1}\mathrm{diag}\left(w_{t}\right)\right]}^{-1}F\right]}^{-1}, \end{array}$$

(27c) $$\begin{array}{c} \;\;B⪰Z^{T}{\left[F+\zeta^{-1}\mathrm{diag}\left(w_{t}\right)\right]}^{-1}Z, \end{array}$$

(27d) $$\begin{array}{c} \;\;K_{t}^{\min}\leq 1^{T}w_{t}\leq K_{t}^{\max}, \end{array}$$

where we introduce the auxiliary variables $V\in S^{N}$ and $B\in S^{N}$. We notice that the epigraph form (27) is well-posed since by choosing $0<\alpha <\sigma_{e}^{2}$ in (17) the matrix $\left[F+\zeta^{-1}\mathrm{diag}\left(w_{t}\right)\right]$ is always positive definite and symmetric. In addition, also the matrix $\left[X-F{\left[F+\zeta^{-1}\mathrm{diag}\left(w_{t}\right)\right]}^{-1}F\right]$ is also positive definite by construction as derived in (18)–(21).

The epigraph form (27) is not a strictly convex program, in the sense that there are multiple V and B matrices that achieve the minimal cost value. This is due to the inequality constraints of (27b) and (27c). At optimality, the eigenvalues of V and B must be equivalent to their lower bounds in (27b) and (27c). Hence, an optimizer of the problem is $V={\left[X-F{\left[F+\zeta^{-1}\mathrm{diag}\left(w_{t}\right)\right]}^{-1}F\right]}^{-1}$ and $B=Z^{T}{\left[F+\zeta^{-1}\mathrm{diag}\left(w_{t}\right)\right]}^{-1}Z$.

We proceed by simplifying the constraint (27b). Let us introduce another auxiliary variable $A\in S^{N}$ and substitute (27b) with two constraints

(28) $$\begin{array}{cc} V & ⪰{\left[X-A\right]}^{-1}, \end{array}$$

(29) $$\begin{array}{cc} A & ⪰F{\left[F+\zeta^{-1}\mathrm{diag}\left(w_{t}\right)\right]}^{-1}F. \end{array}$$

With this in place, the optimization Problem (27) can be formulated as

(30a) $$\begin{array}{c} \underset{w_{t}\in {\left[0,1\right]}^{N},V,A,B\in S^{N}}{arg min}\;\;\mathrm{tr}\left[V\right]+\mathrm{tr}\left[B\right]+\lambda_{t}{∥w_{t}∥}_{1} \end{array}$$

(30b) $$\begin{array}{c} \;s.t.\;V⪰{\left[X-A\right]}^{-1}, \end{array}$$

(30c) $$\begin{array}{c} \;A⪰F{\left[F+\zeta^{-1}\mathrm{diag}\left(w_{t}\right)\right]}^{-1}F, \end{array}$$

(30d) $$\begin{array}{c} \;\;B⪰Z^{T}{\left[F+\zeta^{-1}\mathrm{diag}\left(w_{t}\right)\right]}^{-1}Z, \end{array}$$

(30e) $$\begin{array}{c} \;\;K_{t}^{\min}\leq 1^{T}w_{t}\leq K_{t}^{\max}, \end{array}$$

It can be claimed that the optimization problem (30) is equivalent to (27) given that it yields a decision variable $w_{t}$ with the same optimal cost of (27) . To prove this, let us choose an arbitrary $w_{t}$ say $\bar{w}$. For a fixed yet arbitrary $\bar{w}$ verifying (30e), the optimization problem (30) minimizes both V and B. This means that due to (30b) it minimizes also A: in fact, as $V⪰{\left[X-A\right]}^{-1}$ the lower bound for V is minimal if the positive definite matrix [X−A] is maximal, that is A is minimal. Therefore, A must attain its lower bound. As mentioned earlier, there are multiple optimizers, yet one is $A=F{\left[F+\zeta^{-1}\mathrm{diag}\left(w_{t}\right)\right]}^{-1}F$. In addition, $V={\left[X-A\right]}^{-1}={\left[X-F{\left[F+\zeta^{-1}\mathrm{diag}\left(\bar{w}\right)\right]}^{-1}F\right]}^{-1}$ at optimality, as well. The same reasoning holds also for B, which at optimality is $B=Z^{T}{\left[F+\zeta^{-1}\mathrm{diag}\left(\bar{w}\right)\right]}^{-1}Z$. Since this reasoning is valid for any feasible $\bar{w}$, it is also valid for an optimal one and therefore the equivalence claim follows. It should be noted that a similar argument was also presented in [24], where only the issue of correlated measurement noise is considered.

Using the Schur complement lemma the constraints (30b) and (30c) can be equivalently represented by the linear matrix inequalities (LMI):

(31a) $$\begin{array}{c} \left[\begin{array}{cc} X-A & I\\ I & V \end{array}\right]⪰0 \end{array}$$

(31b) $$\begin{array}{c} \left[\begin{array}{cc} A & F\\ F & F+\zeta^{-1}\mathrm{diag}\left(w_{t}\right) \end{array}\right]⪰0 \end{array}$$

The constraint (30c) can be equivalently represented by an LMI using the Schur complement [28]. In other words, using the fact that $[F+\zeta^{-1}\mathrm{diag}\left(w_{t}\right)]≻0$, we obtain

(32) $$\left[\begin{array}{cc} B & Z^{T}\\ Z & F+\zeta^{-1}\mathrm{diag}\left(w_{t}\right) \end{array}\right]⪰0.$$

Finally, an SDP representation of the overall optimization problem of (27) can be expressed as

(33a) $$\begin{array}{c} \underset{w_{t}\in {\left[0,1\right]}^{N},A,B,V\in S^{N}}{arg min}\;\mathrm{tr}\left[V\right]+\mathrm{tr}\left[B\right]+\lambda_{t}{∥w_{t}∥}_{1}, \end{array}$$

(33b) $$\begin{array}{cc} \; & s.t.\;\;\;\;\mathrm{LMIs}\;\;\mathrm{in}\;\;(31a),\;(31b),\;(31c) \end{array}$$

(33c) $$\begin{array}{cc} \; & \;\;K_{t}^{\min}\leq 1^{T}w_{t}\leq K_{t}^{\max} \end{array}$$

The solution of the aforementioned SDP is a selection vector ${\hat{w}}_{t}\in {\left[0,1\right]}^{N}$.

### 3.2. Spatial Sensor Placement for Stationary Field Estimation

Let us consider the effect of the stationary component of the field $s_{t}$ for any *t*. In this case, we consider that $v_{t}=0$. In this case, the measurement model of (1) is given by $y_{t}=C_{t}s_{t}+e_{t}$. Exploiting the prior information regarding $s_{t}$, i.e., $s_{t}∼N\left(\mu_{s},\Sigma_{s}\right)$ an LMMSE estimator of $s_{t}$ can be presented by ${\hat{s}}_{t}=\mu_{s}+\Sigma_{s}C_{t}^{T}{\left(C_{t}\Sigma_{s}C_{t}^{T}+\sigma_{e}^{2}I_{M_{t}}\right)}^{-1}\left(y_{t}-C_{t}\mu_{s}\right)$. The performance of the aforementioned estimator is given by the MSE matrix $M_{s_{t}}={\left[\Sigma_{s}^{-1}+\sigma_{e}^{-2}C_{t}^{T}C_{t}\right]}^{-1}=\Sigma_{s}-\Sigma_{s}C_{t}^{T}{\left(C_{t}\Sigma_{s}C_{t}^{T}+\sigma_{e}^{2}I_{M_{t}}\right)}^{-1}C_{t}\Sigma_{s}$. Considering the fact that $\Sigma_{s}$ can be ill-conditioned, following the formulation of (23), the expression of $M_{s_{t}}$ can be expressed as a function of $w_{t}$ as

(34) $$\begin{array}{c} M_{s_{t}}=Y+Z^{T}{\left[F+\zeta^{-1}\mathrm{diag}\left(w_{t}\right)\right]}^{-1}Z, \end{array}$$

where $Y=\Sigma_{s}-\Sigma_{s}\Sigma_{\mathrm{sr}}^{-1}\Sigma_{s}$, $Z=\Sigma_{\mathrm{sr}}^{-1}\Sigma_{s}$, and $F=\Sigma_{\mathrm{sr}}^{-1}$. Note that the matrices F, Y, and Z are all independent of $w_{t}$. Considering $g\left(w_{t}\right)=\mathrm{tr}\left[M_{s_{t}}\right]$ and following the same SDP formulation of Section 3.1, the proposed sensor placement problem of (9) can be represented as

(35a) $$\begin{array}{c} \underset{w_{t}\in {\left[0,1\right]}^{N},B\in S^{N}}{arg min}\;\mathrm{tr}\left[B\right]+\lambda_{t}{∥w_{t}∥}_{1}, \end{array}$$

(35b) $$\begin{array}{c} \;s.t.\;\left[\begin{array}{cc} B & Z^{T}\\ Z & F+\zeta^{-1}\mathrm{diag}\left(w_{t}\right) \end{array}\right]⪰0, \end{array}$$

(35c) $$\begin{array}{c} \;\;\;K_{t}^{\min}\leq 1^{T}w_{t}\leq K_{t}^{\max}. \end{array}$$

The optimization problem of (35) gives the spatial sensor placement pattern for any snapshot *t*, when the field is stationary over space. However, if the field is also temporally stationary then the sensor placement problem of (35) can be extended to blocks of multiple snapshots. In this case, the performance metric can be computed using the same approach as [22]. In the simulation section, we show the effects of spatial correlation on sensor placement.

### 3.3. Sparsity-Enhancing Iterative Design

In order to eschew the effect of the magnitude dependencies of the elements of ${\hat{w}}_{t}$, we individually weigh each element of $w_{t}$. In this case, we consider a vector form for the regularization parameter : $\lambda_{t}\in R^{N}$. The weight associated to the each element of $w_{t}$ is the corresponding element of $\lambda_{t}\in R^{N}$. We iteratively refine the weighting vector $\lambda_{t}$ in the $\ell_{1}$ minimization term of the problem (33) [29]. Using this approach, higher weights are applied on the smaller elements of $w_{t}$ to push them towards 0 and the magnitudes of the larger elements are maintained by applying a smaller weight. In this way, a sparser solution can be obtained compared to the standard sparsity-promoting method. The iterative algorithm can be summarized as

- **Initialize**i=0, weight vector $\lambda_{t}^{0}=1_{N}$, ϵ, and maximum number of iterations *I*.
- **for**i=0,…,I
  (36a) $$\begin{array}{c} {\hat{w}}_{t}^{i}=\underset{w_{t}\in {\left[0,1\right]}^{N},A,B,V\in S^{N}}{arg min}\mathrm{tr}\left[V\right]+\mathrm{tr}\left[B\right]+{\left(\lambda_{t}^{i}\right)}^{T}w_{t}, \end{array}$$
  (36b) $$\begin{array}{cc} \; & s.t.\;\mathrm{LMIs}\;\;\mathrm{in}\;\;(31a)\;,(31b),\;(31c) \end{array}$$
  (36c) $$\begin{array}{cc} \; & \;\;K_{t}^{\min}\leq 1^{T}w_{t}\leq K_{t}^{\max} \end{array}$$
- ${\left[\lambda_{t}^{i+1}\right]}_{j}=\frac{1}{\epsilon +{\left[{\hat{w}}_{t}^{i}\right]}_{j}}$, for every j=1,…,N
- **end**;
- **set**${\hat{w}}_{t}={\hat{w}}_{t}^{I}$.

After solving the above algorithm, we still obtain ${\hat{w}}_{t}\in {\left[0,1\right]}^{N}$. We convert this to a Boolean selection vector $w_{t}\in {\left\{0,1\right\}}^{N}$ using a deterministic/stochastic rounding method as mentioned in Section 3.1.

### 3.4. KKF Algorithm with Sensor Placement

The informative $M_{t}$ locations to deploy/move the sensors at any *t* is denoted by $\mathrm{supp}({\hat{w}}_{t})$, where $1^{T}{\hat{w}}_{t}=M_{t}$. The noisy measurements collected from the aforementioned $M_{t}$ locations are stored in $y_{t}$. The sensing matrix $C_{t}$ is constructed by removing the all-zero rows of $\mathrm{diag}({\hat{w}}_{t})$ at every *t*. This measurement matrix is used for the estimation of the non-stationary and the stationary components by (11) and (14), respectively. Then the overall field estimate at time *t* is computed by ${\hat{u}}_{t}={\hat{v}}_{t}+{\hat{s}}_{t}$. Note that the estimation steps, i.e., (11) and (14) do not require the computation of the inverse of $\Sigma_{s}$. The error covariance of the non-stationary component can be updated by (13), which also does not require the inverse of $\Sigma_{s}$. At every *t*, the overall estimation performance can be computed by the expression of (24). The best case performance, i.e., the performance with all the locations selected can also be computed by the expression of (24) by using $w_{t}=1_{N}$.

In many practical environmental fields (such as rainfall), the field is generally non-negative. To achieve a non-negative estimate at every *t*, the estimates of the stationary and non-stationary components can be projected onto the non-negative orthant, i.e., the negative values are set to zero. This is obtained by adopting

(37) $${\hat{u}}_{t}={\left[{\hat{v}}_{t}+{\hat{s}}_{t}\right]}_{+}.$$

However, in this case, the performance metrics $\mathrm{tr}[M_{v_{t}}]$ and $\mathrm{tr}[M_{s_{t}}]$ are only the approximations. The overall sensor placement followed by a KKF algorithm is presented in Algorithm 1.

**Algorithm 1** Sensor placement followed by a KKF estimator.
1:**Initialize**: t=0, ${\hat{v}}_{t}$, $M_{{\hat{v}}_{t}}$, $\lambda_{t}^{0}=1_{N}$.2:**Given**: ${\left\{H_{t}\right\}}_{t=1}^{T}$, $Q_{t}=Q$, $\Sigma_{s}$, $\sigma_{e}^{2}$, *I*, ϵ, $K_{t}^{\max}$, $K_{t}^{\min}$.3:
**for** t=1,…,T
4:    **Prediction using the state model.**5:    **Sensor placement:** iterative sparsity-enhancing design of $w_{t}\in {\left\{0,1\right\}}^{N}$ (Section 3.3).6:    **Correction:** estimation of $v_{t}$ and $s_{t}$ using the measurements from the selected sensing locations.7:    **Overall KKF estimate:** ${\hat{u}}_{t}={\left[{\hat{v}}_{t}+{\hat{s}}_{t}\right]}_{+}$.8:    **Covariance update.**9:
**end for**



## 4. Simulation Results

**Figure 3:**
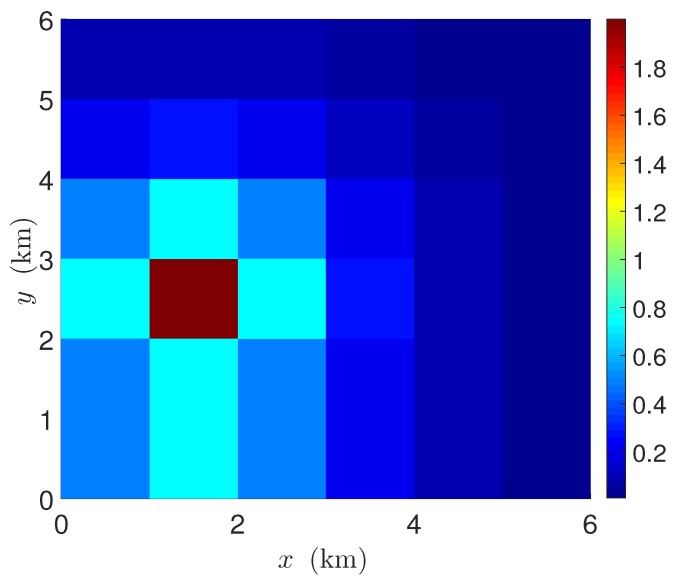
Field distribution at t=0 with a single source: K=1, $\rho_{1}={\left[1.5,1.5\right]}^{T}$, $s_{1}=2$, $d_{1}=1$.

In this section, we perform some numerical experiments to exhibit the practicality of the developed sparsity-enforcing sensor placement followed by the KKF estimation method. We select a service area of 6×6 square km with 1 square km spatial resolution as illustrated in Figure 3. The spatial distribution of the non-stationary component at time t=0, i.e., $v_{0}$, is generated by the following exponential source-field model

(38) $${\left[v_{0}\right]}_{j}=\sum_{k=1}^{K}s_{k}\exp(-d_{k}∥x_{j}-\rho_{k}{∥}_{2}),\;j=1,\ldots ,N,$$

where *K* is the number of field-generating points/sources. The parameters $\rho_{k}$, $s_{k}$, and $d_{k}$ are the location, amplitude, and the spatial decaying factor of the *k*-th source at time t=0. Based on this function, we generate the non-stationary component of the field at time t=0, i.e., $v_{0}\in R^{N}$ using the parameters K=1, $\rho_{1}={\left[1.5,1.5\right]}^{T}$, $s_{1}=2$, and $d_{1}=1$. The spatial distribution of $v_{0}$ in the specified service area is shown in Figure 3.

The state model of the non-stationary component $v_{t}$ is modelled by (7). The state transition matrix is modelled by the Gaussian kernel function given by (8). For the sake of simplicity, we consider a spatially invariant translation parameter and spatio-temporally invariant dilation parameters given as $a_{t}^{ij}=a_{t}$ and $D_{t}^{ij}=D$, respectively, for i,j=1,…,N. The elements of the state transition matrix are given by

(39) $${\left[H_{t}\right]}_{ij}=\nu \exp\left[-{\left(x_{i}-x_{j}-a_{t}\right)}^{T}D^{-1}\left(x_{i}-x_{j}-a_{t}\right)\right].$$

The spatio-temporal evolution of the true value of the field, i.e., $u_{t}=v_{t}+s_{t}$ is generated in the following two ways.

**Figure 4:**
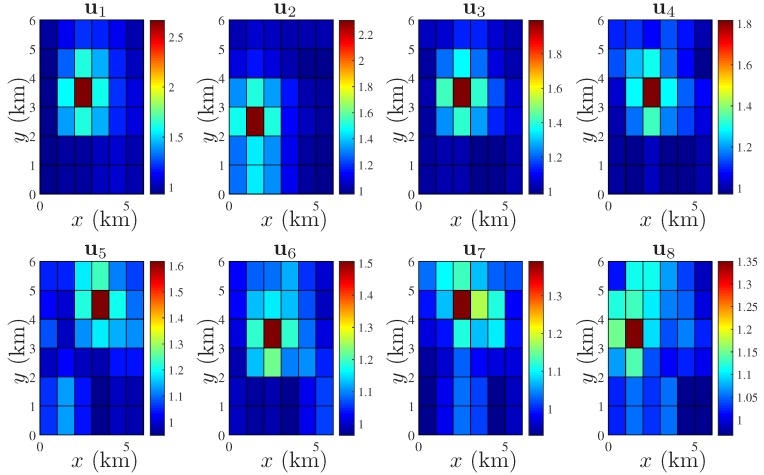
Spatio-temporal evolution of $u_{t}$ in a 6×6 square km area; spatial resolution: 1×1 square km; time varying $H_{t}$ for t=1,…,8; strength of spatial correlation: θ=1.

In the **first** case, we consider a pure advective process, i.e., we select a very low dilation parameter given by $D=10^{-4}I_{2}$ for all t=1,…,8 and ν=0.8. It is assumed that the temporal resolution is 1 min. The translation vectors, i.e., $a_{t}$, are assumed to be changing every *t* as ${\left[1,1\right]}^{T}$, ${\left[-1,-1\right]}^{T}$, ${\left[1,1\right]}^{T}$, ${\left[0,0\right]}^{T}$, ${\left[1,1\right]}^{T}$, ${\left[-1,-1\right]}^{T}$, ${\left[0,1\right]}^{T}$, and ${\left[-1,-1\right]}^{T}$. It is assumed that at t=0, $v_{t}$ is generated by the source as shown in Figure 3. The different states of $v_{t}$ for t=1,…,8 are generated by the state model of (7). The spatially colored yet temporally uncorrelated process noise is characterized by $q_{t}∼N\left(0_{N},Q\right)$, where ${\left[Q\right]}_{ij}=10^{-4}\exp(-∥x_{i}-x_{j}{∥}_{2})$. The stationary component $s_{t}$ is modelled by $s_{t}∼N\left(1_{N},\Sigma_{s}\right)$. The parameters of the squared exponential covariance function of (6) are given by $\sigma_{s}^{2}=0.001$ and θ=1. Note that increasing the value of θ, the field becomes spatially more correlated and the condition number of $\Sigma_{s}$ increases. However, as mentioned earlier, our proposed formulation, i.e., both the selection and the estimation, does not involve the inversion of $\Sigma_{s}$. A highly spatially correlated $s_{t}$ is considered in the next case. For the first case, the true field $u_{t}=v_{t}+s_{t}$ for t=1,…,8 can be simulated as shown in Figure 4.

**Figure 5:**
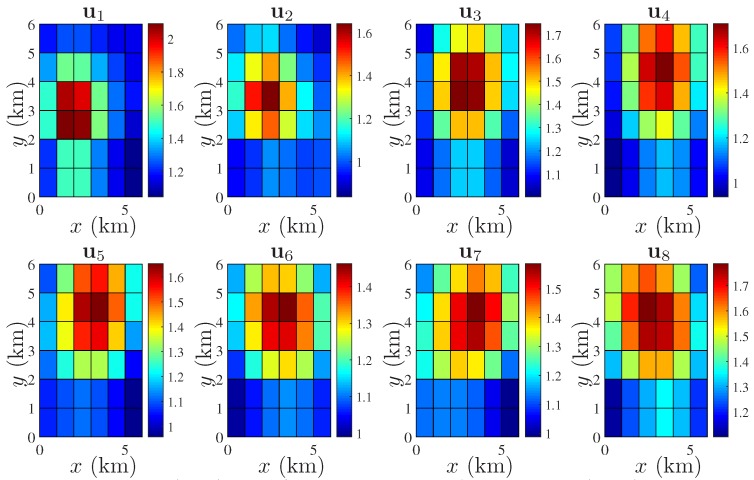
Spatio-temporal evolution of $u_{t}$ in a 6×6 square km area; spatial resolution: 1×1 square km; time varying $H_{t}$ for t=1,…,8; strength of spatial correlation: θ=4.

In the **second** case, we consider $D=I_{2}$ for all t=1,…,8 and the translation parameters are fixed as $a_{t}={\left[0.4,0.4\right]}^{T}$ for t=1,…,4, and no translation for the last 4 snapshots, i.e., $a_{t}={\left[0,0\right]}^{T}$ for t=5,…,8. The state of $v_{t}$ at t=0 is the same as before. The scaling parameter is given by ν=0.35. The process noise $q_{t}$ is the same as before. In this case, we assume that the stationary component $s_{t}$ is spatially more correlated than the last case. The parameters of the covariance Function (6) are taken as $\sigma_{s}^{2}=0.01$ and θ=4, which generates an ill-conditioned $\Sigma_{s}$ (Figure 2a). Using these, the true field, i.e., $u_{t}=v_{t}+s_{t}$ for t=1,…,8 is shown in Figure 5.

### 4.1. Sensor Placement Followed by Field Estimation Using KKF

We select the optimal sensing locations and use them to estimate the field for t=1,…,8 snapshots for the two different scenarios of the spatio-temporal evolution of the field, as mentioned in the previous section. We use the same service area shown in Figure 1b, where the centroids of the N=36 pixels are the candidate sensing locations. We assume that the measurement noise variance is given by $\sigma_{e}^{2}=0.001$. We solve the optimization problem of (36) with the parameters I=2 and $\epsilon =10^{-6}$. The weighting vectors are initialized as $\lambda_{0}=1_{N}$. The resource constraints are given as $K_{t}^{\max}=30$ and $K_{t}^{\min}=25$ for all *t*. To extract the Boolean solution $w_{t}\in {\left\{0,1\right\}}^{N}$ from ${\hat{w}}_{t}\in {\left[0,1\right]}^{N}$, we adopt the randomized rounding method. We use the software CVX [30] (parser CVX, solver SeDuMi [31]) to solve the SDP problem (36).

**Figure 6:**
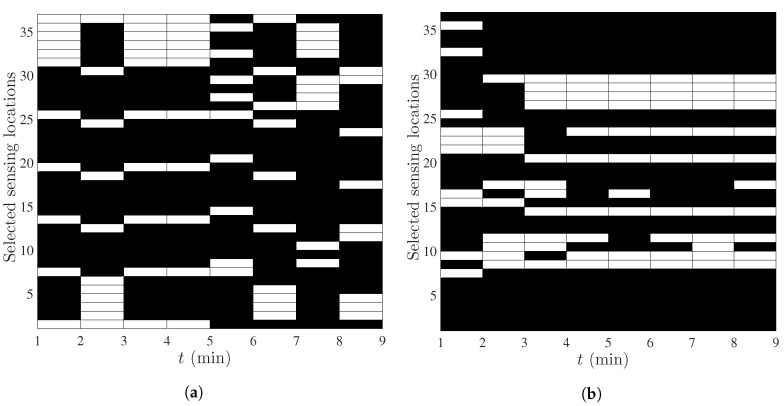
(**a**) Selected sensing locations to estimate the field with the first scenario of the true value; (**b**) Selected sensing locations to estimate the field with the second scenario of the true value.

Following the above simulation setup, the selected sensing locations for the first and the second scenario are shown in Figure 6a,b respectively for the 8 snapshots. The indices of the pixel midpoints are the same as in Figure 1b (vertical axis). The main observations from the selected locations are listed below.

- First of all, it is clearly seen that the selected sensing locations depend on the dynamics. Note that Figure 6a gives the optimal placement pattern, when $H_{t}$ is changing every *t* (different $a_{t}$ on every *t*). Figure 6b shows the optimal sensing locations when we have the same $H_{t}$ for t=1,…,4 ($a_{t}={\left[0.4,0.4\right]}^{T}$) and another $H_{t}$ for t=5,…,8 ($a_{t}={\left[0,0\right]}^{T}$).
- When $H_{t}$ is changing every *t*, i.e., the spatio-temporal evolution of the field is guided by the time-varying spatial translation parameter $a_{t}$ (Figure 1b), the optimal selection pattern also depends upon this translation (Figure 6a).
- In the second scenario, we have assumed a very low and fixed translation, i.e., $a_{t}={\left[0.4,0.4\right]}^{T}$ for the first 4 snapshots and $a_{t}={\left[0,0\right]}^{T}$, i.e., no translation, for the last 4 snapshots (Figure 5). It is seen that almost the same set of sensors are selected in the last 4 snapshots of Figure 6b. In general, when $H_{t}$ is not changing with time, the estimation error of the non-stationary component reaches a steady state after a number of snapshots and the same set of sensors are selected every *t*.

**Figure 7:**
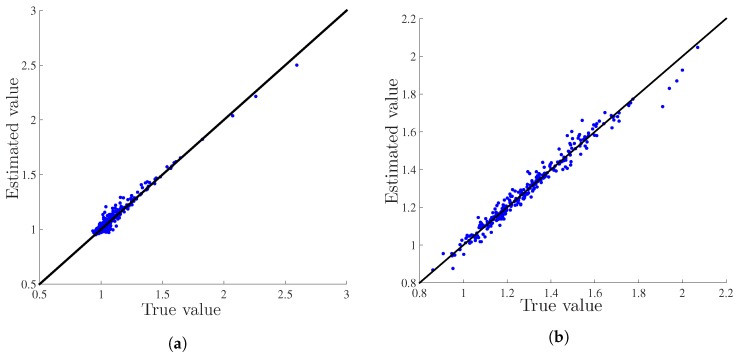
(**a**) Comparison of the KKF estimate (${\hat{u}}_{t}$) with the true value ($u_{t}$) (Figure 4) with the measurements from the selected locations shown in Figure 6a; (**b**) Comparison of the KKF estimate (${\hat{u}}_{t}$) with the true value ($u_{t}$) (Figure 5) with the measurements from the selected locations shown in Figure 6b.

The overall estimation performance using the measurements from the selected locations of Figure 6a,b is shown in Figure 7a,b, respectively. In these figures, we exhibit the pixel-wise comparison of the estimates for T=8 snapshots, i.e., the estimation performance of 36×8=288 pixels. We initialize the KKF iterations with ${\hat{v}}_{t}=1_{N}$ and $M_{v_{t}}=0.001I_{N}$ at t=0.

### 4.2. Performance Analysis

**Figure 8:**
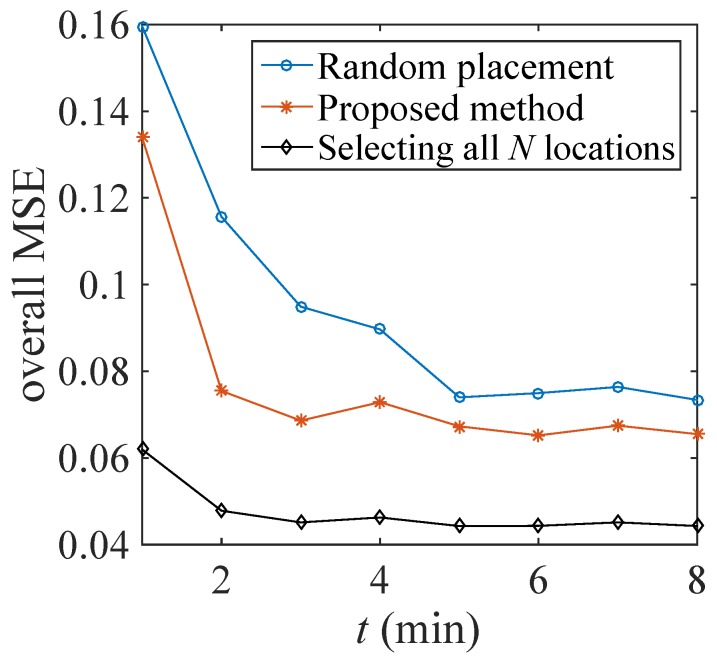
Comparison of the performance metric for the proposed method, random placement and the best case (only 15 sensors are selected on every *t*).

We compare the estimation performance of the developed sensor placement method by comparing the performance metric, i.e., $g\left({\hat{w}}_{t}\right)=\mathrm{tr}\left[M_{s_{t}}\right]+\mathrm{tr}\left[M_{v_{t}}\right]$ with a random sensor placement (with the same $M_{t}$, i.e., $∥{\hat{w}}_{t}{∥}_{0}=M_{t}$ as for the developed method) and with the best case performance (i.e., $M_{t}=N$ or $w_{t}=1_{N}$). For the random placement, we generate 100 different realizations of $w_{t}\in {\left\{0,1\right\}}^{N}$ at every *t* with the same $M_{t}$ as for the proposed method. Then $g(w_{t})$ is computed for every $w_{t}$ and their average is considered. Similarly, we compute the best case performance, i.e., $g(1_{N})$ for every *t* and in this case $M_{v_{t}}$ is updated with $w_{t}=1_{N}$. We use the same set of parameters as mentioned in the first case of Section 4.1. Only the resource allocation constraint is simplified as $1^{T}w_{t}=15$, i.e., we fix that only 15 sensing locations will be selected every *t*. The performance comparison is shown in Figure 8. It is seen that the proposed approach slightly outperforms the random placement. However, the random placement of sensors does not optimize any performance criterion.

### 4.3. Spatial Sensor Placement for Stationary Field Estimation

**Figure 9:**
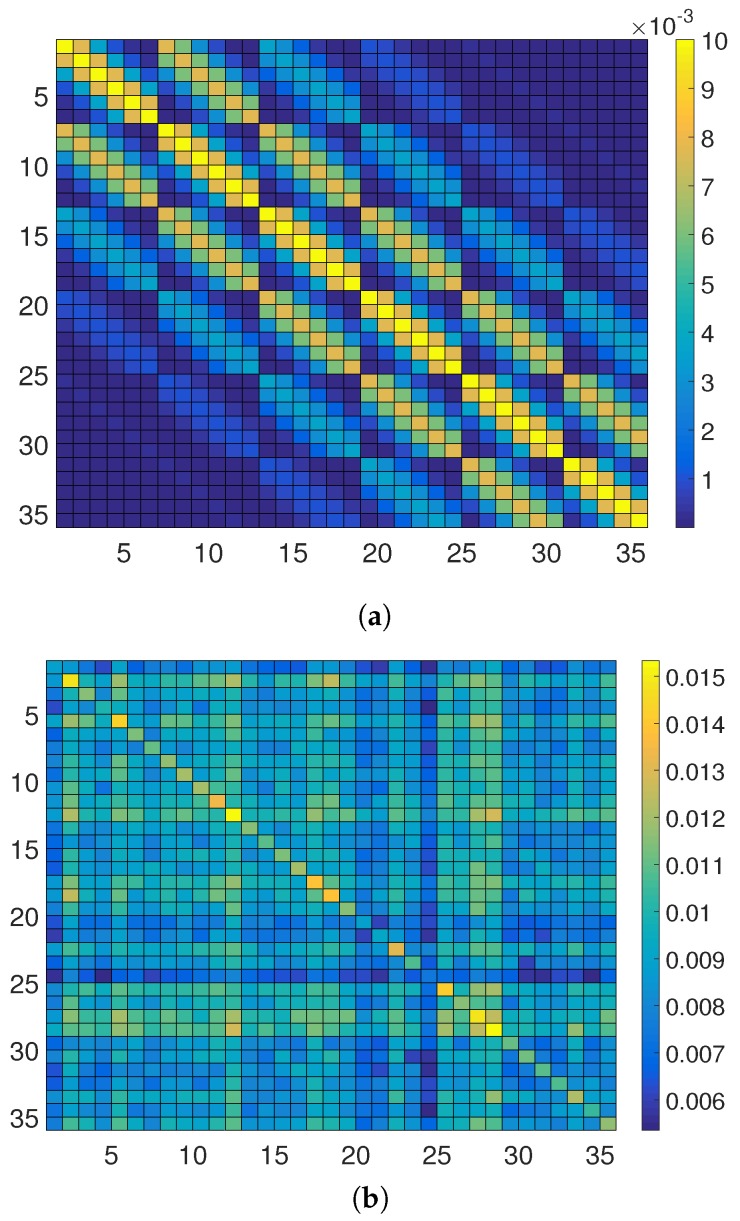
(**a**) Spatial covariance matrix generated by the squared exponential function ($\sigma_{s}^{2}=0.01,$θ=2); (**b**) Randomly generated spatial covariance matrix.

**Figure 10:**
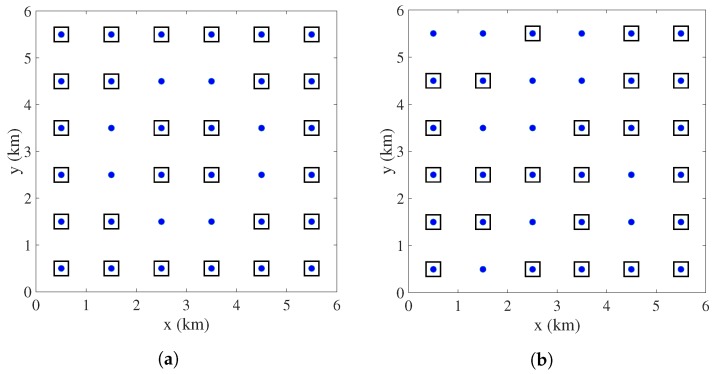
(**a**) Sensor placement pattern for the $\Sigma_{s}$ as shown in Figure 9a; (**b**) Sensor placement pattern for the $\Sigma_{s}$ as shown in Figure 9b.

In this section, we show the effects of different spatial correlation patterns on sensor placement assuming the field is purely stationary. We solve the optimization problem of (35), for two different spatial covariance matrices ($\Sigma_{s}$). In the first case, we consider that $\Sigma_{s}$ is generated by the squared exponential Function (6) with θ=2 and $\sigma_{s}^{2}=0.01$ (Figure 9a). In the second case, we consider a randomly generated $\Sigma_{s}$ (Figure 9b). The resource allocation constraint is the same as before, i.e., $K_{t}^{\min}=25$, and $K_{t}^{\max}=30$. We solve the optimization problem of (35), with the iterative approach of (36) with the same parameters as mentioned in the previous section. The selected locations (marked by black squares where the blue dots are the candidate locations as shown in Figure 1b) to deploy sensors are shown in Figure 10a,b for the spatial covariance matrices shown in Figure 9a,b, respectively.

First of all, it is observed that the spatial distribution of the optimal sensing locations depends upon the correlation pattern of the field. It is seen that when $\Sigma_{s}$ is generated by a squared exponential covariance (stationary) function then the optimal sensor placement pattern is more or less symmetrically and uniformly distributed over the entire service area. However, for a random spatial covariance matrix the optimal sensing locations do not follow any specific pattern.

## 5. Conclusions and Future Work

In this work, we have developed a sparsity-enforcing sensor placement followed by a field estimation technique using a KKF. The proposed methodology selects the most informative sensing locations over space and time in a specified service area of interest. Along with minimizing the estimation error, the developed method also economizes the sensor placement (in terms of resources) at every temporal interval. The salient features of the proposed method include handling a general class of spatial covariance matrices and tackling correlated measurement noise. Numerical analysis shows the feasibility of the method. The effects of the dynamics and spatial correlation of the field in spatio-temporal sensor placement are discussed with numerical experiments.

In this work, we have considered the fact that the prior knowledge regarding the spatial variability and the dynamics are perfectly known a priori. In that case, the performance of a clairvoyant Kalman setup with Gaussian measurement and process noise is optimal. However, in many practical scenarios, the aforementioned spatio-temporal prior information may not be accurate and we require more information regarding the unknown field in the estimation step. Future research is envisioned to incorporate the effects of model imperfections in the developed method. Another future research area could be using distributed algorithms to apply the developed method for large scale sensor network applications. It will also be interesting to tailor the recent progress in time-varying optimization [32] to solve the SDPs in a tracking fashion, rather than at optimality at each sampling time.

## References

[1] Oliveira L., Rodrigues J. (2011). Wireless sensor networks: A survey on environmental monitoring. J. Commun..

[2] Hart J.K., Martinez K. (2006). Environmental sensor networks: A revolution in the earth system science?. Earth-Sci. Rev..

[3] Cressie N., Wikle K. (2011). Statistics for Spatio-Temporal Data.

[4] Mardia K.V., Goodall C., Redfern E.J., Alonso F.J. (1998). The kriged Kalman filter. Test.

[5] Wikle C.K., Cressie N. (1999). A dimension-reduced approach to space-time Kalman filtering. Biometrika.

[6] Kim S.J., Anese E.D., Giannakis G.B. (2011). Cooperative spectrum sensing for cognitive radios using kriged Kalman filtering. IEEE J. Sel. Top. Signal Process..

[7] Rajawat K., Dall’Anese E., Giannakis G. (2014). Dynamic network delay cartography. IEEE Trans. Inf. Theory.

[8] Sahu S.K., Mardia K.V. (2005). A Bayesian kriged Kalman model for short term forecasting of air pollution levels. J. R. Stat. Soc. Ser. C (Appl. Stat.).

[9] Cortes J. (2009). Distributed Kriged Kalman filter for spatial estimation. IEEE Trans. Autom. Control.

[10] Krause A., Singh A., Guestrin C. (2008). Near-optimal sensor placements in Gaussian processes: Theory, efficient algorithms and empirical studies. J. Mach. Learn. Res..

[11] Ranieri J., Chebira A., Vetterli M. (2014). Near-optimal sensor placement for linear inverse problems. IEEE Trans. Signal Process..

[12] Carmi A. Sensor scheduling via compressed sensing. Proceedings of the 13th Conference on Information Fusion (FUSION).

[13] Fu Y., Ling Q., Tian Z. (2012). Distributed sensor allocation for multi-target tracking in wireless sensor networks. IEEE Trans. Aerosp. Electron. Syst..

[14] Patan M. (2012). Optimal Sensor Networks Scheduling in Identification of Distributed Parameter Systems.

[15] Liu S., Fardad M., Masazade E., Varshney P.K. (2014). Optimal periodic sensor scheduling in networks of dynamical systems. IEEE Trans. Signal Process..

[16] Mo Y., Ambrosino R., Sinopoli B. (2011). Sensor selection strategies for state estimation in energy constrained wireless sensor networks. Automatica.

[17] Akyildiz I.F., Vuran M.C., Akan O.B. On exploiting spatial and temporal correlation in wireless sensor networks. Proceedings of the WiOpt.

[18] Joshi S., Boyd S. (2009). Sensor selection via convex optimization. IEEE Trans. Signal Process..

[19] Jamali-Rad H., Simonetto A., Ma X., Leus G. (2015). Distributed sparsity-aware sensor selection. IEEE Trans. Signal Process..

[20] Chepuri S.P., Leus G. (2015). Sparsity-promoting sensor selection for non-linear measurement models. IEEE Trans. Signal Process..

[21] Liu S., Cao N., Varshney P.K. Sensor placement for field estimation via poisson disk sampling. Proceedings of the 2016 IEEE Global Conference on Signal and Information Processing (GlobalSIP).

[22] Roy V., Simonetto A., Leus G. (2016). Spatio-temporal sensor management for environmental field estimation. Signal Process..

[23] Jindal A., Psounis K. (2006). Modeling spatially correlated data in sensor networks. ACM Trans. Sens. Netw..

[24] Liu S., Chepuri S.P., Fardad M., Maşazade E., Leus G., Varshney P.K. (2016). Sensor selection for estimation with correlated measurement noise. IEEE Trans. Signal Process..

[25] Ababou R., Bagtzoglou A.C., Wood E.F. (1994). On the condition number of covariance matrices in kriging, estimation, and simulation of random fields. Math. Geol..

[26] Sigrist F., Künsch H.R., Stahel W.A. (2012). A dynamic nonstationary spatio-temporal model for short term prediction of precipitation. Ann. Appl. Stat..

[27] Kay S.M. (1993). Fundamentals of Statistical Signal Processing: Estimation Theory.

[28] Boyd S., Vandenberghe S. (2009). Convex Optimization.

[29] Candes E., Wakin M., Boyd S. (2008). Enhancing sparsity by reweighted *ℓ*_1_ minimization. J. Fourier Anal. Appl..

[30] Grant M., Boyd S., Ye Y. (2008). CVX, Matlab Software for Disciplined Convex Programming.

[31] Sturm J.F. (1999). Using sedumi 1.02, a matlab toolbox for optimization over symmetric cones. Optim. Methods Softw..

[32] Simonetto A., Dall’Anese E. (2017). Prediction-correction algorithms for time-varying constrained optimization. IEEE Trans. Signal Process..

